# Syndromic Surveillance for Influenza in the Emergency Department–A Systematic Review

**DOI:** 10.1371/journal.pone.0073832

**Published:** 2013-09-13

**Authors:** Katherine M. Hiller, Lisa Stoneking, Alice Min, Suzanne Michelle Rhodes

**Affiliations:** Department of Emergency Medicine, University of Arizona, Tucson, Arizona, United States of America; National Institutes of Health, United States of America

## Abstract

The science of surveillance is rapidly evolving due to changes in public health information and preparedness as national security issues, new information technologies and health reform. As the Emergency Department has become a much more utilized venue for acute care, it has also become a more attractive data source for disease surveillance. In recent years, influenza surveillance from the Emergency Department has increased in scope and breadth and has resulted in innovative and increasingly accepted methods of surveillance for influenza and influenza-like-illness (ILI). We undertook a systematic review of published Emergency Department-based influenza and ILI syndromic surveillance systems. A PubMed search using the keywords “syndromic”, “surveillance”, “influenza” and “emergency” was performed. Manuscripts were included in the analysis if they described (1) data from an Emergency Department (2) surveillance of influenza or ILI and (3) syndromic or clinical data. Meeting abstracts were excluded. The references of included manuscripts were examined for additional studies. A total of 38 manuscripts met the inclusion criteria, describing 24 discrete syndromic surveillance systems. Emergency Department-based influenza syndromic surveillance has been described worldwide. A wide variety of clinical data was used for surveillance, including chief complaint/presentation, preliminary or discharge diagnosis, free text analysis of the entire medical record, Google flu trends, calls to teletriage and help lines, ambulance dispatch calls, case reports of H1N1 in the media, markers of ED crowding, admission and Left Without Being Seen rates. Syndromes used to capture influenza rates were nearly always related to ILI (i.e. fever +/− a respiratory or constitutional complaint), however, other syndromes used for surveillance included fever alone, “respiratory complaint” and seizure. Two very large surveillance networks, the North American DiSTRIBuTE network and the European Triple S system have collected large-scale Emergency Department-based influenza and ILI syndromic surveillance data. Syndromic surveillance for influenza and ILI from the Emergency Department is becoming more prevalent as a measure of yearly influenza outbreaks.

## Introduction

Influenza is a perennial disease with the potential for yearly outbreaks. Approaches to surveillance of influenza activity and influenza-like-illness (ILI) in the community have traditionally been multifactorial, taking data from sentinel providers, such as primary care physicians, laboratory data, hospital admission rates and mortality data into account. These data sources, while important, historically lag one to two weeks behind real-world outbreaks. Primary care physicians and laboratories typically only operate and report a fixed number of hours in the day. In addition, uninsured or underinsured patient populations, may not have a primary care physician from whom they can seek care. Acute and non-lethal influenza in this population may only be evaluated by emergency providers. The emergency department (ED) is open 24 hours a day, 7 days a week, and has the potential to capture a wider variety of patients, especially those who are not able to seek care elsewhere.

The science of surveillance is rapidly evolving due to changes in public health information and preparedness as national security issues, new information technologies and health reform. [1] Syndromic surveillance is a relatively new field of study that has its roots in bioterrorism surveillance. [2], [3] It is attractive as a surveillance method in part because it is efficient, sensitive and near real-time. [4], [5] A framework for evaluating syndromic surveillance systems for the early detection of outbreaks has been established by the Centers for Disease Control and Prevention (CDC). [6] In general, surveillance systems should be timely and complete, should improve the ability to recognize patterns indicative of possible disease outbreaks early in their course, and frequently use new types of data such as product purchase, work or school absenteeism, presenting symptoms, or laboratory test orders. These recommendations have been adapted for use by ambulatory care networks and focused for the purpose of influenza surveillance into six criteria: (1) data collected should exist for reasons other than bioterrorism surveillance, (2) data should be electronically recorded and accessible, (3) data should be available near-real-time, (4) there should be sufficient historical data to adequately describe a definable population, (5) syndromes should be validated against existing traditional data sources, and (6) thresholds set for the system should be sensitive and provide a high positive predictive value. [7].

The CDC and the World Health Organization (WHO) have relied on a network of sentinel providers and laboratories across the United States in order to estimate the effect of seasonal and pandemic influenza (http://www.cdc.gov/flu/weekly/fluactivitysurv.htm). Reports of ILI by primary care physicians, pneumonia and influenza mortality data, and laboratory reports of positive influenza tests currently provide the foundation for influenza surveillance. The only published review on syndromic surveillance, though focused on bioterrorism, did identify 13 systems of syndromic surveillance for influenza in 1998. None of the systems identified incorporated data derived from the ED. [3] Traditionally, the ED has played a relatively minor role in surveillance for influenza; however, as the ED has become a much more utilized venue for acute care, it has also become a more attractive data source for disease surveillance. In recent years, influenza surveillance from the ED has increased in scope and breadth and has resulted in innovative and increasingly accepted methods of surveillance for influenza.

We present a systematic review of syndromic surveillance for influenza and ILI in the emergency department. The objective of this review is to synthesize the methods used for syndromic surveillance for influenza and ILI throughout the world, and to describe the use of syndromic surveillance for influenza specifically from the ED setting. While syndromic surveillance certainly can be and has been used in other venues, the ED represents a large patient population, frequently not captured elsewhere, from which to extrapolate data. Similarly, syndromic surveillance for diseases other than influenza has been effectively utilized; however, influenza is a unique and perennial disease entity that provides a yearly opportunity for system testing. Finally, syndromic surveillance is but one of the multiple methods used for surveillance. At the minimum it can augment other data sources such as laboratory or mortality data, and it may be more sensitive and faster than other surveillance data, especially when ED data are used.

## Methods

We undertook a systematic review of published ED-based influenza syndromic surveillance systems. A Pubmed search of all published work using the keywords “syndromic”, “surveillance”, “influenza” and “emergency” was performed at the onset of the project, in April of 2012, and again in April 2013. The search was not limited by language, publication type, or any other criteria. All abstracts recovered were reviewed by all four study personnel for the following inclusion criteria: the abstract described a syndromic surveillance system for which (1) patients presented to an Emergency Department, (2) clinical data (fever, ILI, seizure, gastrointestinal complaints, etc) or visit data (number of visits, ambulance calls, etc) were used for surveillance, and (3) at least one of the diseases under surveillance was influenza. Full text manuscripts were reviewed for all abstracts when all four investigators agreed that the study met inclusion criteria, or when there was a split decision. Abstracts that all four investigators felt did not meet the inclusion criteria were not included. Full text manuscripts were reviewed by an investigator for the same inclusion criteria listed above. If the manuscript clearly met or did not meet the inclusion criteria, it was included or excluded as appropriate. If inclusion criteria were not clearly met on the first investigator’s read, a second investigator also reviewed the paper. If there was subsequently a split decision between the two reviewers, a consensus decision was made among all investigators. After the initial medical literature search and review, the references of included manuscripts were reviewed for potential manuscripts that might meet the inclusion criteria in addition to those uncovered with the original search. A second round of abstract review/paper review and cross-referencing was performed, using the same inclusion criteria as described above. See PRISMA Flow Diagram.

Full text manuscripts that met study criteria were reviewed for the following data elements: (1) description of the population studied (location, urban/rural, large/small community, pediatric/all ages), (2) description of the institution (location, academic/community, multi-center/single-center), (3) the syndrome used (ILI, fever, respiratory complaint, etc), (4) how the syndrome was determined (presenting complaint, physician diagnosis, triage diagnosis, billing, discharge, combination), (5) pediatric versus adult ED, and (6) linkage to confirmatory or otherwise validated surveillance systems.

Upon review of included studies, it was clear that some of the established syndromic surveillance systems had published more than one manuscript. In these cases, we included all the manuscripts that met our inclusion criteria. Excluded manuscripts that did not meet our inclusion criteria, but were based on the same system, were used to better understand data collection and validation processes if such information was not clear in the original included work.

Once the included manuscripts were finalized, a descriptive analysis of data collected was reported. Specifically, descriptions of the populations under surveillance including. geography, age range, and institution from which the surveillance originated are presented. In addition, descriptive analysis of the ED data used for surveillance and linkage data (if any) is reported.

A review of published full-length manuscripts inherently contains the risk of publication bias, namely, there are likely small-scale syndromic surveillance projects or projects initiating from health agencies, military or other government-contracted groups that have been carried out in EDs that did not result in full-length publication. While academic EDs likely publish data related to such work, community and governmental projects may have been missed with this strategy.

## Results

A total of 58 studies were reviewed from the original Pubmed search. Of these, 41 full text manuscripts were reviewed, and 30 met the inclusion criteria. Cross-referencing abstracts from the included manuscripts generated a total of 56 additional abstracts, of which 31 non-duplicated papers were reviewed and 8 met inclusion criteria. This yielded a total of 38 manuscripts for inclusion which described 24 discrete syndromic surveillance systems. The most frequent reasons for exclusion were that the surveillance was not performed in the ED (i.e. data used for surveillance was generated from an urgent care or clinic setting), influenza was not among the diseases under surveillance, or the paper was a description of statistical methods related to a syndromic surveillance system already included. The included manuscripts are presented in Table S1.

Syndromic surveillance for influenza and ILI has been described in EDs worldwide. Countries that have published descriptions of their syndromic surveillance systems include: the United States, Canada, Australia, Italy, China, Guam, Taiwan, Israel, France, and the United Kingdom. Systems included in our review varied with regard to scope and geographic area covered. Some were limited to a single ED or small population center, [8] and some were multinational, collating data from multiple large, urban population centers and vast geographic areas. [9] While most of the systems reviewed were initiated at an academic center, many encompassed community EDs as well. Both pediatric and adult EDs have participated in syndromic surveillance.

A wide variety of clinical data was used for surveillance (see Table S1). The most frequently used data were chief complaint or ED presentation [9], [10], [11], [12], [13], [14], [15], [16], [17], [18], [19], [20], [21], [22], [23], [24], [25], [26], [27], [28], [29], [30], [31], [32], [33], [34], [35], [36], [37], [38], [39], [40], [41] and preliminary or discharge diagnosis codes [8], [9], [11], [16], [17], [18], [22], [23], [26], [27], [32], [33], [38], [39], [41], [42]. Other creative data used to capture influenza activity included free text analysis of the entire ED medical record, [37] Google flu trends, [25] calls to teletriage and help lines, [16], [25], [38] ambulance dispatch calls, [19], [20], [21], [30], [31], [32] case reports of H1N1 in the media, [8] ED census/”saturation”/length-of-stay data, [36] and admission and Left Without Being Seen (LWBS) rates. [36].

Syndromes used to capture influenza rates were nearly always related to ILI (i.e. fever +/− a respiratory or constitutional complaint); however, other syndromes used for surveillance included fever alone [34], [35] and seizure. [32], [33], [41].

The observed syndromic cases based on ED data were in some cases linked to objective, confirmatory data, such as culture and other laboratory results, [8], [10], [11], [12], [14], [15], [16], [17], [18], [19], [20], [21], [24], [25], [27], [30], [31], [32], [33], [34], [35], [37], [41] and in some cases to traditional regional and national surveillance databases [9], [13], [16], [17], [22], [25], [26], [27], [29], [40], [43], historic data [44] and pneumonia and influenza weekly mortality data [12], [14].

Two of the studies reviewed established or evaluated new ED influenza surveillance systems specifically during large, multinational events, the Rugby World Cup in Sydney [28] and the 2012 London Olympic and Paraolympic games. [45].

Some programs utilized the same ED information system for all centers within the system. This allowed for uniform and consistent data reporting. Some large systems that collected data from diverse and remote sites were able to collect and aggregate data from varied sources in different formats.

In the United States, multiple metropolitan areas have reported on ED surveillance systems. In 2006, the International Society for Disease Surveillance (ISDS) developed the DiSTRIBuTE (Distributed Surveillance Taskforce for Real-time Influenza Burden Tracking and Evaluation) network, a project that allowed collaboration between 10 jurisdictions across North America, constructing a very large and diverse ED influenza syndromic surveillance system. [9] This system was structured to rely on existing state and local systems and expertise and allowed individual centers to report aggregate data in their own native format. The program was greatly expanded in 2009 when the program partnered with the Centers for Disease Control and Prevention (CDC) BioSense system. At its height, DiSTRIBuTE had over 50 centers and it is estimated that over 40% of all US ED visits were captured through the DiSTRIBuTE system, making it the largest syndromic surveillance system worldwide. This allowed for not only more comprehensive and complete ED syndromic surveillance for influenza, but also for research and program evaluation on best practice paradigms for ED surveillance for influenza in general. [46], [47], [48], [49], [50], [51] The DiSTRUIBuTE program ceased data collection in May 31, 2012, due to “the evolving nature of syndromic surveillance in the US.” (http://syndromic.org/component/content/article/11-programming/35-distribute-project, accessed April 24, 2013).

In Europe, the Triple S system (Syndromic Surveillance Systems in Europe) began on September 1, 2010 as a three year project coordinated by the French Institute for Public Health Surveillance (InVS). [52] It “encompasses an inventory of existing and proposed syndromic surveillance systems in Europe.” (http://syndromicsurveillance.eu/, accessed April 24, 2013) While the system has yet to specifically publish on influenza surveillance from the ED, several recent poster presentations at national and international meetings report on its progress. Triple S members include emergency medicine and EMS providers, and it collects data on influenza and influenza-like-illness.

## Discussion

There are a number of syndromic surveillance systems for influenza and ILI in use in EDs across the world. International influenza surveillance from the ED occurs in North America, Europe, Africa and Asia. In the United States, of the top five population centers according to the 2010 United States census (NYC, Los Angeles, Chicago, Houston and Philadelphia), two have reported data from ED-based influenza surveillance systems. [19], [20], [21], [30], [31], [36].

While chief complaint and discharge diagnosis data are the most often used data from the ED encounter, many researchers have used other, creative data sources for surveillance, including telephonic triage calls, ED volume, LPTMS rates, and ambulance dispatch data. ILI is the most often used syndrome used to identify surveillance cases, however novel syndromes, such as febrile seizures in the pediatric population, unexplained pneumonia, or even more broad clinical complaints such as fever alone, have been used to estimate community levels of influenza.

Though not necessary for a surveillance system, many of the reported systems linked their surveillance data to other objective data, such as viral culture, PCR or other laboratory data. Frequently, the systems described were compared with other, more established surveillance systems not based in the ED, such as regional or national surveillance systems.

## Limitations

Our initial review focused on studies indexed in PubMed. We spent considerable time cross-referencing original articles identified to ensure that those not readily identified by our original search would be included. Despite this measure, it is possible that our initial search strategy missed one or more manuscripts which were not indexed in PubMed. This may disproportionately affect the reporting of syndromic surveillance systems in non-English speaking countries.

As with any systematic review, our study suffers from the risk of publication bias. In our data collection phase, we reviewed several abstracts that seemed to describe influenza syndromic surveillance systems. Studies that have been presented at a scientific meeting but not published may represent a very real source of bias in the systematic review process. There are likely other academic investigators who have studied whether syndromic surveillance for influenza is feasible in their ED, however, we felt that such small-scale work, while foundational, is not truly representative of the state of syndromic surveillance for influenza in the global emergency medicine community. Additionally, governmental projects, either public health or military based, may not be published in the scientific literature. While this represents a significant limitation, we did find that this type of surveillance was at least obliquely identifiable by reference in the included manuscripts. Of 99 abstracts on syndromic surveillance presented at the 2003 National Syndromic Surveillance Conference hosted by the CDC, 40% of authors were based in state and local health departments, 32% universities, 13% federal governmental agenies, 11% health care organizations, and 4% businesses. [53].

While we did not explicitly search for online descriptions of systems operated by local, state, federal or international health agencies, when such systems were cited in a manuscript we did review them. One example of a large-scale system that did not meet our inclusion criteria, but was referenced in more than one included manuscript is the Triple S program. It is currently collecting influenza syndromic data from EDs in Europe, but has not yet published results of their system as related to this particular disease and clinical venue. (http://syndromicsurveillance.eu/, accessed April 24, 2013). Another example of a project which did not meet our inclusion criteria, but which has been referenced by other work is the ESSENCE II surveillance system. The ESSENCE II project used syndromic surveillance for influenza and ILI by reviewing ambulatory care records for TriCare, the US military’s health-care system and additionally focused on non-traditional health-care indicators, including civilian ED chief-complaint records. [54], [55], [56].

## Conclusions

The ED is a valuable setting for syndromic surveillance of influenza and ILI and may be more sensitive and faster than traditional surveillance methods. Syndromic surveillance for influenza and ILI from the ED is becoming more prevalent and more accepted as a measure of yearly influenza outbreaks. As the ED continues to become a more and more utilized health care setting, the acceptability and validity of syndromic surveillance from the ED will continue to improve.

## Supporting Information

Figure S1 **PRISMA Flow Diagram.**
(TIF)

Table S1 Included studies of ED-based influenza syndromic surveillance systems.
(DOCX)

Checklist S1 **PRISMA Checklist.**
(DOC)
