# Supplementary figures and images for: Syndromic Surveillance for Influenza in the Emergency Department–A Systematic Review
Source: PLoS One. 2013 Sep 13;8(9):e73832. doi: 10.1371/journal.pone.0073832 (PMC3772865; doi:10.1371/journal.pone.0073832)

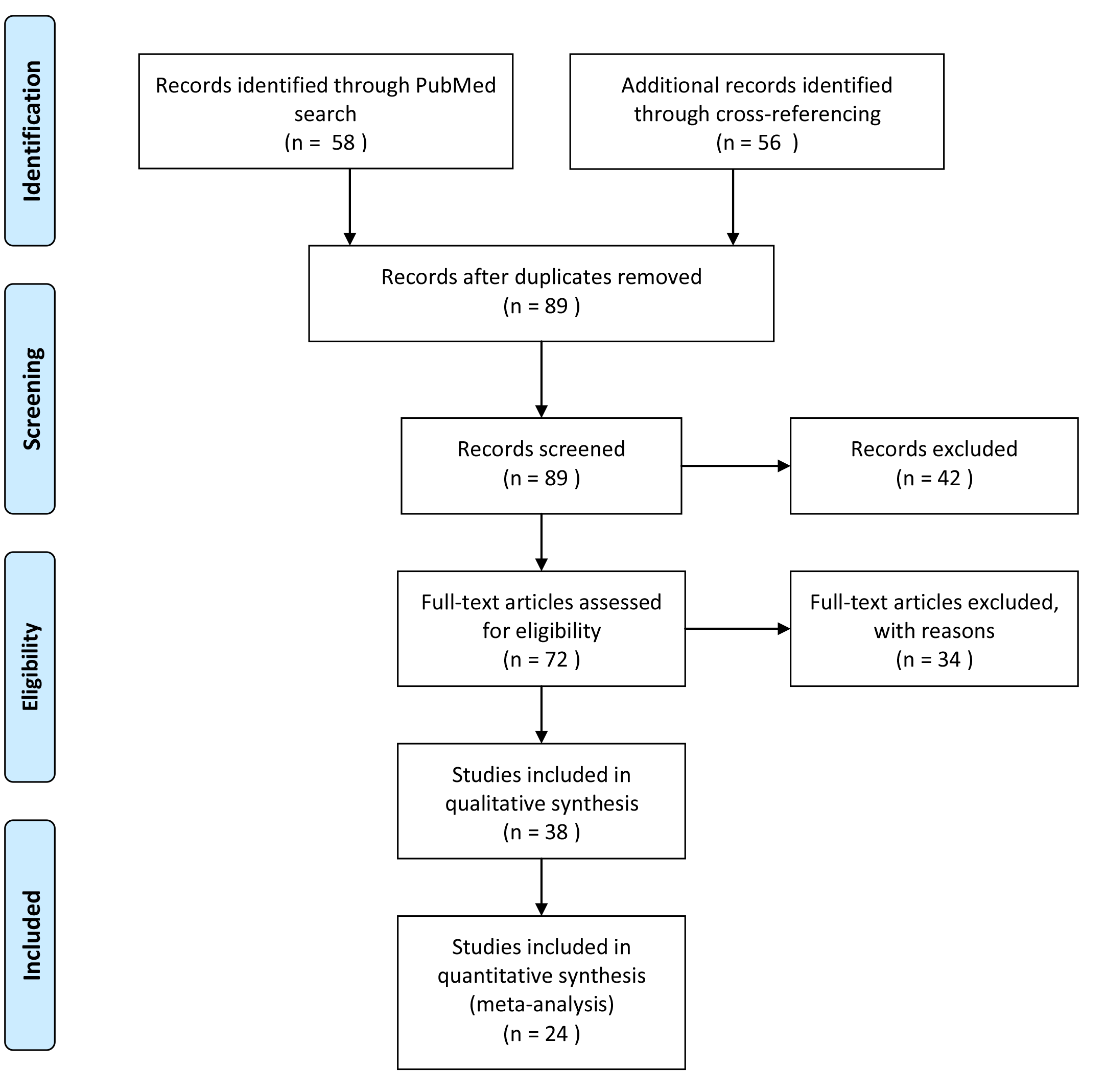

Supplement: Figure S1 — PRISMA Flow Diagram. (TIF) [file pone.0073832.s001.tif]
