# Supplementary material for: Syndromic Surveillance for Influenza in the Emergency Department–A Systematic Review
Source: PLoS One. 2013 Sep 13;8(9):e73832. doi: 10.1371/journal.pone.0073832 (PMC3772865; doi:10.1371/journal.pone.0073832)
Supplement: Table S1 — Included studies of ED-based influenza syndromic surveillance systems. (DOCX) [file pone.0073832.s002.docx]

**Table 1. Included studies of ED-based influenza syndromic surveillance systems**

| **Location** | **Population/ Institution** | **Syndrome** | **Data analyzed** | **Linkage to dependent data** | **Notes** |
| --- | --- | --- | --- | --- | --- |
| Sydney, New South Wales, Australia [[32](#_ENREF_32),[33](#_ENREF_33)] [[41](#_ENREF_41)] | 35 EDs in NSW, EMS dispatch database | Seizure, ILI (and 5 other resp syndromes), provisional flu dx | ED presentation (ICD-9 or -10 codes) | ILI weekly rates; Lab | [[28](#_ENREF_28)]describes system r.e. Rugby Cup |
| Melbourne, Australia [[27](#_ENREF_27)] | Tertiary care hospital, 2 EDs in Melbourne | Presenting ICD-10 codes for influenza | Presenting symptoms (free text) and prelim dx (ICD-10) | Victorian DOH ID system, Lab. | [[29](#_ENREF_29)] describes system |
| Wellington New Zealand [[42](#_ENREF_42)] |  |  | ICD10 discharge codes |  |  |
| Genoa Italy [[15](#_ENREF_15)] [[10](#_ENREF_10)] | Regional Reference University Hospital | ILI  LRTI | Keywords in specific fields (anamnesis, hx, exam, comments); ED chief complaint | Lab |  |
| Paris, France [[22](#_ENREF_22)] | 46 EDs: 31 in Paris, 14 other regions France, 1 Indian Ocean | ICD-10 codes for influenza | CC, + ICD-10 diagnosis codes (from free text) | Sentiweb (national sentinel network) |  |
| Canada [[25](#_ENREF_25)] | Canadian multicenter EDs | ILI | CC | Lab; Google flu trends, FluWatch (national network) |  |
| Ontario Canada [[38](#_ENREF_38)] | 7 community hospitals | “Respiratory” | CC | Calls to teletriage; discharge ICD-10 codes |  |
| Ottawa Canada [[24](#_ENREF_24)] | 3 local hospitals | Fever, cough, resp illness, HA, ST, myalgias | CC--keywords | Lab |  |
| Israel [[34](#_ENREF_34)] | ED serving pop of 250,000 | Fever, influenza | CC fever | Lab | [[35](#_ENREF_35)] |
| Wuxi, China [[44](#_ENREF_44)] | Multicenter | ILI | Data from hospital-based outpatient clinics and EDs | Compared to retrospective data |  |
| Taiwan [[39](#_ENREF_39)] | 189 hospitals | ILI | CC, ICD-9 diagnosis codes |  |  |
| US [[9](#_ENREF_9)] | Multiple state/local EDs | “preferred local syndrome grouping used by the (local) health department…” | Weekly aggregate data from all sites based on native syndromic criteria. | ILINet |  |
| Guam [[8](#_ENREF_8)] | Civilian ED in Guam | “acute respiratory infection” | “swine flu” in local media; ED diagnosis | Lab |  |
| Madison, WI [[11](#_ENREF_11)] [[18](#_ENREF_18)] | 1 ED, Madison WI | ILI syndrome definitions/diagnostic codes | ED CC & diagnosis at presentation/discharge; ICD-9 codes | Lab |  |
| Washington DC [[26](#_ENREF_26)] | Single, urban, academic ED | CC: fever, HA, dry cough, ST, rhinorrhea, myalgias.  Dx: bronchitis, pneumonia, URI, acute sinusitis, pharyngitis, fever, myalgias. | Relationship of CC and diagnosis for the syndromes. | CDC ILI rates. |  |
| King County/Seattle [[23](#_ENREF_23)] | 18 EDs in King County | ILI | CC, discharge dx and case reports of H1N1 | Lab, case reports |  |
| Baltimore, MD and SLC, UT [[37](#_ENREF_37)] | VA hospitals (ED and outpt) | ILI | CC, ED notes, nursing notes/triage vs entire text | Multiple MD-reviewers |  |
| Boston, MA [[12](#_ENREF_12)] [[14](#_ENREF_14)] | ED of a tertiary care children’s hospital | Resp illness/  infectious resp illness, 2 of: F, cough, sneezing, ST, runny nose, congestion | CC; free text converted to 181 possible dx (17=resp) | Lab, mortality data |  |
| SE Virginia; Hampton Roads/Tidewater region [[40](#_ENREF_40)] | 7 VA hospitals; “significant military presence” | 7 syndromes (fever, respiratory distress, vomiting, diarrhea, rash, disorientation and sepsis) | CC categorized into 1 of 7 syndromes. On high occurrence days, chart reviews | local sentinel surveillance system (CDC) |  |
| NYC, US [[20](#_ENREF_20),[21](#_ENREF_21)] [[19](#_ENREF_19)] [[30](#_ENREF_30)] [[31](#_ENREF_31)] | 39 EDs, EMS dispatch | Respiratory, fever, diarrhea, vomiting | Free text CC, EMS dispatch data | Lab, ED dx ILI |  |
| Los Angeles, CA [[36](#_ENREF_36)] | Urban, teaching hospital | Flu index: fever, infection, or respiratory CC | CC “fever/infection” or “respiratory” (also ED census, LOS, admission rate, total bed time, LWBS, saturation) | CDC data for “widespread” activity |  |
| Wash, D.C. [[43](#_ENREF_43)] | Military ED | Respiratory syndrome | Standardized ambulatory data record (discharge dx) | CDC data for ILI |  |
| Pittsburg, PA [[16](#_ENREF_16),[17](#_ENREF_17)] | U of Pittsburgh Medical Center Health System EDs, telephone triage | Acute resp illness (<5 days of cough, SOB, sputum, abnormal lung exam or XR w/ PNA); Telephone triage guidelines ‘respiratory’ and ‘constitutional’ | CC and discharge dx; Which telephonic triage guideline was followed | CDC weekly regional flu activity, ILI, Lab. |  |
| Los Alamos, NM [[13](#_ENREF_13)] | UH | “respiratory” Uses ESSENCE/RODS categories [[56](#_ENREF_56)] | CC | Statewide sentinel flu system |  |
